# Editorial: Immune thrombocytopenia (ITP) - diagnosis and treatment, volume II

**DOI:** 10.3389/fmed.2025.1756865

**Published:** 2026-01-12

**Authors:** Tomás José González-López

**Affiliations:** Department of Hematology, Hospital Universitario de Burgos, Burgos, Spain

**Keywords:** diagnosis, immune, ITP, thrombocytopenia, treatment

Immune thrombocytopenia (ITP) is characterized by an abnormally reduced platelet count in peripheral blood, arising from a dysregulated immune response that compromises both platelet production and survival. Recent evidence indicates that, beyond classical antibody-mediated platelet destruction, this disease encompasses defects in cytotoxic lymphocyte–mediated injury, perturbations in mesenchymal stem-cell immunoregulatory functions, activation of apoptotic pathways in platelets (caspase 3, 8, and 9 activation and phosphatidylserine exposure) and expansion of T follicular helper (Tfh) cells. However, despite numerous diagnostic advances, ITP is principally identified through the systematic exclusion of other potential etiologies.

The main goal of ITP treatment is to prevent and control bleeding by maintaining platelet counts within safe hemostatic limits. Advances in the understanding of ITP pathophysiology have facilitated the development of several novel ITP therapies. Nevertheless, considerable obstacles remain in both the accurate diagnosis and effective management of ITP, underscoring the need for a more comprehensive understanding of the disease.

This overview highlights key challenges in the diagnosis and management of ITP. Diagnostic considerations include here the emerging links of ITP with gut microbiome alterations, the dynamic relationship between platelet counts and psychological state after COVID-19 infection as well as disease characteristics of ITP in adolescents and young adults. This ITP volume also discusses therapeutic considerations that may affect the outcomes of corticosteroids and thrombopoietin receptor agonists (TPO-RAs), particularly in patients with comorbidities.

Khiabani et al. conducted a systematic review to evaluate evidence on gut microbiome alterations in ITP. After screening 480 records, 12 studies comparing ITP patients with controls were included, revealing notable but inconsistent changes across major bacterial phyla. Several taxa and microbial metabolites were linked to immune mechanisms involved in platelet destruction and impaired production. Overall, the review suggests a potential association between gut dysbiosis and ITP, though heterogeneity among studies limits firm conclusions and highlights the need for standardized, well-designed research.

Schifferli reviewed ITP in adolescents and young adults (AYAs), a population situated between pediatric and adult ITP and often overlooked in clinical guidance. Drawing on three registry datasets, the review outlines newly diagnosed, chronic, refractory and secondary ITP in individuals aged 12–25 years. AYAs typically present with acute thrombocytopenia resembling childhood disease, yet over half progress to chronic ITP, exhibiting adult-like disease persistence. A smaller group develops refractory disease with lower platelet counts and increased bleeding/treatment burden, while secondary ITP accounts for ~20% of chronic cases, commonly associated with systemic autoimmunity, Evans syndrome or immunodeficiency. The author highlights the need for tailored management approaches that reduce steroid exposure, enable early recognition of secondary causes and prioritize long-term quality of life.

Chen et al. retrospectively analyzed platelet kinetics and psychological responses in 90 ITP patients with a post-Omicron infection compared with 69 healthy controls. Most patients (63%) exhibited a transient post-infection platelet rise, peaking at week 1 and returning to baseline by week 3–4, regardless of baseline platelet level, vaccination or treatment status. Although overall fear scores were comparable to healthy individuals, ITP patients showed greater concern over bleeding risk, delayed platelet recovery and disease worsening. These findings indicate a temporary platelet elevation following COVID-19 and highlight disease-specific anxiety requiring targeted follow-up and psychological support.

Liu et al. conducted a systematic review and network meta-analysis comparing TPO-RAs in adult ITP. Four agents—romiplostim, avatrombopag, eltrombopag and hetrombopag—were analyzed across 14 trials (*n* = 1,454) with additional safety data. Romiplostim showed the highest efficacy (92.9% of responses), followed by avatrombopag and hetrombopag, while eltrombopag ranked lower despite similar effect. Safety differed minimally from placebo, though avatrombopag had the most favorable profile. Nine hundred eighty-two TPO-RA–related events were revealed, mainly linked to romiplostim and eltrombopag, likely due to longer clinical exposure. Overall, treatment choice should be individualized, with romiplostim preferred for maximal response and avatrombopag as a safer option.

Moser et al. conducted an open-label phase II pilot trial to assess whether very low-dose rituximab can sustain CD20+ B-cell depletion in autoimmune hemolytic anemia (AIHA). Ten patients received escalating regimens (5 mg/m^2^, 20 mg, 50 mg, or 100 mg) to evaluate suppression durability and pharmacokinetics. Most achieved ≥95% initial depletion, yet long-term suppression was inconsistent across doses, with only select cases maintaining prolonged reduction. CD20+ cells remained undetectable when rituximab levels exceeded ~0.4 μg/mL, supporting this as an empirical threshold. Overall, very low dosing produces transient but not reliably sustained depletion, indicating a need for further dose-optimization research.

Yu et al. retrospectively assessed whether absolute monocyte count (AMC) at admission could predict relapse-free survival (RFS) in idiopathic thrombotic thrombocytopenic purpura (TTP) after first-episode remission. In 37 patients classified by AMC ranges, elevated AMC (>0.80 × 10^9^/L) correlated with superior RFS, supported by Kaplan–Meier analysis (log-rank *p* = 0.026) and Cox regression as an independent protective factor (Hazard Ratio = 0.12; 95% Confidence Interval = 0.02–0.62). No other hematologic or clinical variables demonstrated prognostic relevance. These results position AMC as a feasible immunologic biomarker for relapse prediction in TTP and a potential tool for guiding post-remission surveillance and therapeutic intensity.

Finally, Beyene et al. performed a cross-sectional study to assess health-related quality of life (HRQoL) and corticosteroid-related complications in ITP across two Ethiopian teaching hospitals. Using the Amharic version of the ITP Life Quality Index in 214 patients, the authors quantified HRQoL burden and explored clinical predictors of impairment. Corticosteroid toxicity was common, with 66.8% reporting adverse effects, predominantly fatigue, emotional disturbances and physical appearance changes. Multivariate analysis linked poorer HRQoL to emotional side effects, fatigue, absence of cotrimoxazole prophylaxis, mucosal bleeding, residence outside the capital of Ethiopia, Addis Ababa, and skin manifestations. These findings underscore the heavy HRQoL impact associated with corticosteroid use and highlight the need for treatment strategies mindful of toxicity.

The contributions collected in this volume explore multiple, and in many cases insufficiently characterized, dimensions of the disease. Advancing research based on these observations will be essential to deepen diagnostic insight and improve therapeutic strategies in ITP.

